# Preoperative serum CA125: a useful marker for surgical management of endometrial cancer

**DOI:** 10.1186/s12885-015-1260-7

**Published:** 2015-05-12

**Authors:** Tao Jiang, Ling Huang, Shulan Zhang

**Affiliations:** Department of Gynecology and Obstetrics, Shengjing Hospital of China Medical University, No.36, Sanhao Street, Heping District, Shenyang, Liaoning Province 110004 China

**Keywords:** CA125, Endometrial cancer, Surgical management

## Abstract

**Background:**

Surgery plays an important role in the management of endometrial cancer at all stages, particularly early clinical stage. There are still many unanswered questions regarding optimal surgical management of endometrial cancer, particularly regarding which patients should undergo lymphadenectomy. The aim of this study was to evaluate the role of preoperative cancer antigen 125 (CA125) serum levels for surgical management in endometrial cancer patients.

**Methods:**

A total of 995 patients with endometrial cancer, according to inclusion criteria of a preoperative serum level of CA125, were selected. The association between clinicopathological factors and CA125 were analyzed. Receiver operating characteristic (ROC) curve was used to evaluate the role of preoperative serum CA125 in predicting lymph node metastasis, adnexal involement, cervical stromal invasion in all patients, especially patients with clinical stage I. Survival analyses were also performed according to the four groups of preoperative CA125 serum levels.

**Results:**

Elevated CA125 level was significantly associated with all clinicopathological parameters, including age and menopause, but not histology type. ROC curve analysis results showed the CA125 serum level of 25 U/mL was the best cutoff to predict the lymph node metastasis. It was with 78% of sensitivity, 78% of specificity, 77.6% of false positive rate, 2.3% of false negative rate in all patients. In patients with clinical stage I, it was with 71.7% of sensitivity, 77.6% of specificity, 83.3% of false positive rate, 2.2% of false negative rate. The best cutoff to evaluate adnexal involement in patients with clinical stage I was 30U/ml, with 81% sensitivity, and 78.4% specificity. Survival analysis revealed CA125, FIGO stage, histology grade, and positive peritoneal cytology as independent prognostic factors of endometrial cancer.

**Conclusion:**

Preoperative serum CA125 is an important predictor for patients with endometrial cancer and it should be taken into consideration when surgical management is determined, especially if a lymphadenectomy should be undertaken in patients with clinical stage I.

## Background

Endometrial cancer is the fourth most frequent cancer in women and the most common gynecological cancer in developed countries. Each year, endometrial cancer develops in approximately 142,000 women worldwide, with an estimated 42,000 deaths from this cancer [1]. The standard treatment of endometrial carcinoma is surgery, including hysterectomy, bilateral salpingo-oophorectomy, pelvic and periaortic lymphadenectomy. Although the uterine cancer staging system changed from a clinical to a surgical system in 1988, and was revised in 2009 by the International Federation of Gynecology and Obstetrics (FIGO), routine usage of pelvic and periaortic lymphadenectomy in the surgical management is still controversial. The disadvantage of systematic lymphadenectomy is a 13-22% risk of lower limb lymphedema after surgery [2,3], along with lymph cyst formation, increased anesthesia and operating time, and the need for a specialized surgical oncologist. Omitting lymphadenectomy in grade 1 or 2 tumors with less than 50% myometrial invasion, the incidence of undiagnosed lymph node metastasis is acceptable for patients with endometrial cancer. However, the most significant hurdle to adopt this system for identifying low-risk disease at the time of surgery is the reliability of frozen section. Accordingly, in the United States, the Gynecologic Oncology Group (GOG) generally requires complete pelvic and periaortic lymphadenectomy in protocols involving clinically early-stage endometrial cancer [4].

The elevation of cancer antigen 125 (CA125) were first described in patients with recurrent and advanced endometrial cancer by Niloff [5] in 1984. Since then, many studies have confirmed that serum CA125 concentrations in patients with endometrial cancer are associated with deep myometrial invasion, extrauterine spread, positive peritoneal cytology, lymph node metastasis, recurrence, advanced stages, and reduced survival [6-12]. However, many of these studies had limitations, such as a small number of patients, and the appropriate reference cutoff values of serum CA125 was inconsistent between these studies, which limited its clinical utility.

Thus, we designed the current study to evaluate the preoperative serum levels of CA125 in patients with endometrial cancer in relation to clinicopathological parameters, and whether these serum levels could provide additional information in determining the extent of surgical management. In particular, we focused on whether preoperative CA125 serum levels could indicate if a lymphadenectomy was required for patients with clinical stage I, and what cutoff value was optimal in this respect.

## Methods

### Patients

The material in our current study was collected from a total of 1,226 patients with endometrial cancer admitted to the Shengjing Hospital of China Medical University from January 2006 to December 2009. This study was approved by the Ethics Committee of the Shengjing Hospital of China Medical University. Blood samples for the analysis of serum CA125 were taken from the patients up to 10 days before surgery. An enzyme immunoradiometric assay with monoclonal antibody was used and the upper normal value of serum CA125 levels were 35 U/mL. All patients received surgical management in our hospital. Patients with disease limited to the uterus received hysterectomy, bilateral salpingo-oophorectomy, ± pelvic and periaortic lymphadenectomy and washing cytology. Patients suspected of having gross cervical involvement received radical hysterectomy, bilateral salpingo-oophorectomy, pelvic ± periaortic lymphadenectomy and washing cytology. Patients with extrauterine disease received complete cytoreductive surgery. If necessary, adjuvant chemotherapy or radiotherapy of the pelvic and periaortic regions was performed depending on the pathological result after the operation according to National Comprehensive Cancer Network (NCCN). The clinical stage were defined according to the 1971 FIGO staging. Pathological stage, histological subtype and lymph node status were defined for each surgical specimen according to the 2009 FIGO criteria. At the end of December 2013, 35 patients had been lost to follow-up. All recurrences were confirmed by radiography, histopathology, or both.

### Inclusion criteria

The inclusion criteria for this study were: patients with histological confirmation of endometrial cancer without history of chemotherapy or radiotherapy; those who underwent complete staging including hysterectomy, bilateral salpingo-oophorectomy, pelvic ± periaortic lymphadenectomy and washing cytology; those whose serum CA125 level was evaluated preoperatively; no pelvic endometriosis or adenomyosis or ovarian primary tumors. According to the inclusion criteria, 231 patients were excluded because of the lack of preoperative serum CA125 levels (n = 121), incomplete staging (n = 32), lack of follow up (n = 35), presentation with endometriosis or adenomyosis or ovarian primary tumors (n = 36) and insufficient data (n = 7). Therefore, a total of 995 patients were enrolled in this study and all gave their informed consent.

### Statistical analysis

Data were analyzed using SPSS statistical software (SPSS, Chicago, IL, USA). The data on serum CA125 levels was not a standard normal distribution, so a nonparametric test was used to evaluate its relation with clinicopathological parameters. The levels of serum CA125 in different group were analyzed using a Mann–Whitney U test and a Kruskal–Wallis H test. Receiver operating characteristic (ROC) curve analysis was used to find a cutoff level of CA125 in serum with optimal diagnostic sensitivity and specificity. Survival analysis was carried out using the Kaplan–Meier estimation and log-rank test. Prognostic factors were assessed using the Cox proportional hazards model. For all analyses, values of *P* < 0.05 were considered significant.

## Results

### Patient characteristic

A total of 995 patients with endometrial cancer were eligible for the study. The mean age was 55.68 ± 9.25 years (20–82 years) and 96.0% of the patients had endometrioid cancer. Of the cases, 35.7% were in the reproductive stage and 64.3% were in the post-menopausal stage. Average duration of menopause among the post-menopausal cases was 12.31 ± 8.47 years (2–30 years), while the average parity was 2.36. The mean body mass index (BMI ) of 995 patients was 26.32 ± 3.94 kg/m^2^. According to the 1971 FIGO staging, the patients with the clinical stage I and II–IV were respectively 864 (86.8%) and 131(13.2%). The mean value of preoperative serum CA125 was 43.6 ± 11.69 U/mL (range 1–1899 U/mL). The number of patients with CA125 > 35 U/mL was 234 (23.5%). The median follow-up period was 64 months (range 3–93 months). In total, 198 patients with recurrence were found in the follow-up period.

### Preoperative serum CA125 and clinicopathological parameters

The results were listed in Table 1. With the exception of histology type, elevated CA125 level was significantly associated with all clinicopathological parameters, including age and menopause. When we evaluated the best cutoff level of clinicopathologic factors using a ROC curve analysis, the CA125 serum levels ranged from 18.25 to 45.08 U/mL with 60.2–86.5% sensitivity, and 43.9–81.7% specificity (Table 2).

**Table 1:** **Association between preoperative serum CA125 and clinicopathological factors**

Characteristic	No of patients	Mean rank of CA125	P value
Age			
≤50	253	530.87	
>50	742	486.79	0.04
Menopause			
No	354	527.53	
Yes	641	481.69	0.02
BMI (kg/m^2^)			
<25	501	517.25	
≥25	494	478.47	0.03
FIGO stage			
I	769	425.27	
II	85	642.04	
III	116	785.00	
IV	25	913.76	0.00
Histology type			
Endometrioid cancer	955	495.08	
Non endometrioid cancer	40	567.83	0.12
Histology grade			
1	495	447.52	
2	367	533.45	
3	133	588.06	0.00
Lymph node metastasis			
no	895	466.82	
yes	100	777.04	0.00
Adnexal involvement			
No	931	473.39	
yes	64	856.01	0.00
Distance metastasis			
no	958	482.37	
yes	37	885.90	0.00
myometrial invasion			
≤1/2	810	456.38	
>1/2	185	674.41	0.00
Cervical stromal invasion			
No	859	460.02	
yes	136	737.92	0.00
Positive peritoneal cytology			
No	964	485.19	
yes	31	909.97	0.00

**Table 2:** **Diagnostic values of preoperative serum CA125 levels for predicting clinicopathological factors in endometrioid endometrial cancer**

	Cutoff of value (U/mL)	AUC	Sensitivity (%)	specificity (%)
Advanced stage (III–IV)	30.00	0.86	80.9	80.0
Grade 3 disease	21.12	0.60	60.2	60.0
> 1/2 myometrial invasion	18.25	0.65	62.8	63.9
Cervical stromal invasion	22.76	0.73	67.1	74.1
Lymph node metastasis	25.00	0.83	78.1	77.5
Positive peritoneal cytology	45.08	0.91	82.9	85.1
Adnexal involvement	30.00	0.90	84.6	84.3
Distance metastasis	41.64	0.91	86.5	83.5

### Preoperative serum CA125 and lymph node metastasis

**Figure 1:**
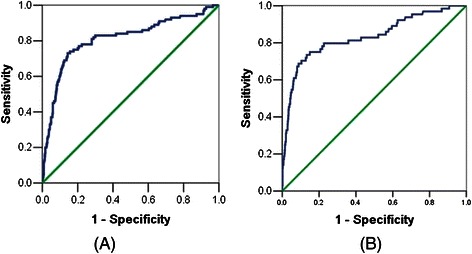
The receiver operating characteristic (ROC) curve of preoperative serum CA125 for predicting lymph node metastasis. **(A)** All patients with lymph node metastasis. **(B)** Patients with only lymph node metastasis without adnexal involvement, distant metastasis and positive peritoneal cytology.

In all patients, the preoperative serum CA125 level of endometrial cancer patients was significantly associated with lymph node metastasis. When we evaluated the best cutoff level of lymph node metastasis factors using ROC curve in all cases, the CA125 serum level of 30 U/mL was found to be best, with 78% sensitivity, 78% specificity, 72.6% false positive rate, and 3.1% false negative rate (Figure 1A). When we used preoperative serum CA125 to predict only lymph node metastasis without adnexal involvement, distant metastasis and positive peritoneal cytology, the CA125 serum level of 25 U/mL was best, with 78% of sensitivity, 78% of specificity, 77.6% of false positive rate, 2.3% of false negative rate (Figure 1B). Considering the influence of age and menopause on CA125 level, we also tested the best cutoff of CA125 in patients with different ages and fertile patients or patients with menopause. In patients aged ≤50 years or in reproductivity, the best cutoff level of serum CA125 was 30 U/mL. In patients aged >50 years or in menopause, the best cutoff level of serum CA125 was also 25 U/mL.

### The role of preoperative serum CA125 in patients with clinical stage I

**Figure 2:**
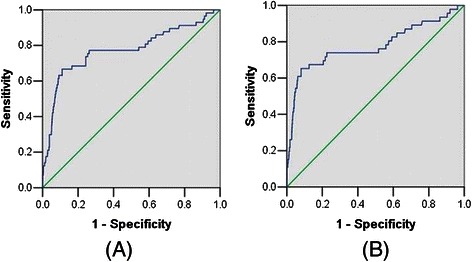
The receiver operating characteristic (ROC) curve of preoperative serum CA125 for predicting lymph node metastasis in patients with clinical stage I. **(A)** All patients with lymph node metastasis. **(B)** Patients with only lymph node metastasis without adnexal involvement, distant metastasis and positive peritoneal cytology.

**Figure 3:**
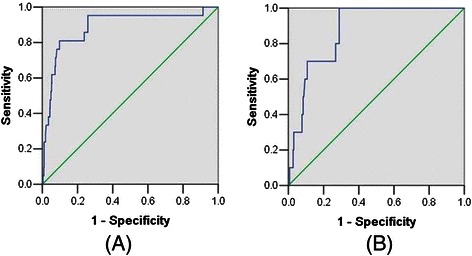
The receiver operating characteristic (ROC) curve of preoperative serum CA125 for predicting adnexal involvement in patients with clinical stage I. **(A)** All patients with adnexal involement. **(B)** Premenopausal patients with adnexal involement.

In 864 patients with clinical stage I, the patients with the FIGO stage I, II, III and IV were respectively 735, 59, 64 and 6. In patients with clinical stage I, the level of CA125 was also related to lymph node metastasis (*P* < 0.01). When we evaluated the best cutoff level of lymph node metastasis factors using a ROC curve in patients with clinical stage I, the CA125 serum level of 25 U/mL was best, with 75.4% of sensitivity, 73.7% of specificity, 83.1% of false positive rate, 2.3% of false negative rate (Figure 2A). When we used preoperative serum CA125 to predict only lymph node metastasis without adnexal involvement, distant metastasis and positive peritoneal cytology, the CA125 serum level of 25 U/mL was also best, with 71.7% of sensitivity, 77.6% of specificity, 83.3% of false positive rate, 2.2% of false negative rate (Figure 2B). The best cutoff to evaluate cervical stromal invasion in patients with clinical stage I was 22U/ml, with 69.7% sensitivity, and 70.4% specificity. The best cutoff value of serum CA125 level of 30 U/mL was with 81% of sensitivity, 78.4% of specificity in predicting adnexal involvement in patients with clinical stage I (Figure 3A).When we focused on premenopausal patients, the best cutoff value of serum CA125 level of 30 U/mL was with 80% of sensitivity, 73.2% of specificity in predicting adnexal involvement (Figure 3B).When we used preoperative serum CA125 to predict extrauterine metastasis, 30 U/mL was the best, with 74.3% of sensitivity and 81.9% of specificity.

### Survival analysis

**Figure 4:**
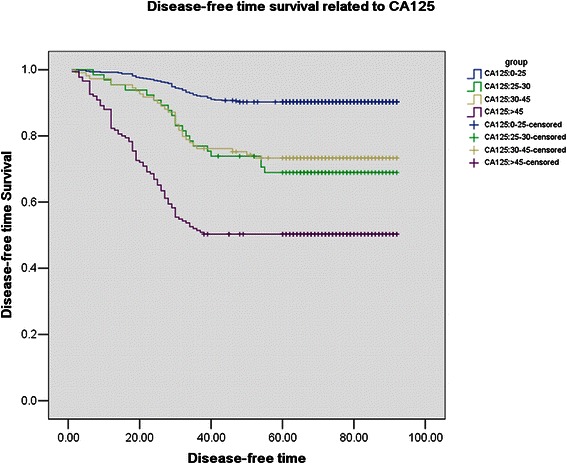
Survival curves in relation to different preoperative serum CA125 group. Prognosis worsened with increasing level of CA125 (χ^2^ = 186.60, P < 0.01).

All patients were divided into four groups according to preoperative serum level of CA125: ≤ 25 U/mL, 25–30 U/mL, 30–45 U/mL, > 45 U/mL. The mean disease-free survival time was 85.75, 72.96, 74.61, 55.56 years for the different groups, respectively (Figure 4). When 12 clinicopathological factors and CA125 were added into the multivariate Cox regression model simultaneously, CA125, FIGO stage, histology grade, and positive peritoneal cytology were also identified as independent prognostic factors (Table 3).

**Table 3:** **Multivariate analysis of prognostic factors for disease-free survival in endometrial cancer**

Factor	No of patients	P value	HR	95.0% CI for HR	
				lower	upper
FIGO stage					
I	769	0.01	1		
II	85	0.15	1.40	0.88	2.20
III	116	0.85	1.04	0.69	1.56
IV	25	0.00	2.34	1.38	3.97
Histology grade					
1	495	0.00	1		
2	367	0.16	1.28	0.91	1.81
3	133	0.00	2.46	1.78	3.42
Positive peritoneal cytology					
No	964		1		
yes	31	0.00	3.24	1.89	5.32
Preoperative serum CA125					
≤25U/mL	645	0.00	1		
25-30 U/mL	65	0.00	2.86	1.71	4.78
30-45 U/mL	109	0.06	1.54	0.98	2.41
>45 U/mL	176	0.00	2.41	1.69	3.46

## Discussion

In the current study, 23.5% of patients with endometrial cancer had > 35 U/mL of serum CA125 levels. This result was similar to previous studies [13-15], which reported that 11–34.9% of patients with endometrial cancer had > 35 U/mL of serum CA125 levels. In addition, 10.05% (n = 100) patients were found to have lymph node metastasis according to the final pathological result in all patients. Furthermore, 6.4% were found to have only lymph node metastasis without adnexal involvement.distance metastasis and positive peritoneal cytology.

Preoperative assessment of lymph node involvement represents a critical step for determining the extent of surgery in patients with endometrial carcinoma, especially in patients with clinical stage I. Interestingly, the mean value of CA125 in the 6.4% patients was significantly higher than those with FIGO stage I. In the current study, the CA125 serum level of 25 U/mL was the best cutoff to determine the lymph node metastasis without influence of adnexal involvement, distant metastasis and positive peritoneal cytology. It had 78% of sensitivity, 78% of specificity, 77.6% of false positive rate, and 2.3% of false negative rate. The incidence of lymph node metastasis with CA125 < 25 U/mL was only 2.3%. However, the incidence of lymph node metastasis in patients with CA125 ≥ 25 U/mL rises to 22.4%. And in clinical stage I patients with endometrial cancer, 25 U/mL was also the best, with 71.7% of sensitivity, 77.6% of specificity, 83.3% of false positive rate, and 2.2% of false negative rate in predicting only lymph node metastasis. In clinical stage I patients, the incidence of lymph node metastasis in patients with CA125 < 25 U/mL was only 2.2%. However, the incidence of lymph node metastasis in patients with CA125 > 25 U/mL rises to 22.7%. In our study, using the Mayo Clinic algorithm for omitting lymphadenectomy to include endometrioid histology, grade 1 or grade 2 tumors, myometrial invasion less than or equal to 50% and no evidence of any metastatic disease at the time of surgery [16], the sensitivity, specificity, false positive and false negative rates are 71.8%, 80.2%, 76.7% and 2.8%, respectively. In patients with low risk, the incidence of lymph node metastasis was 2.8%, but in the patients with high risk the incidence of lymph node metastasis rises to 23.2%. It is worth mentioning that while the data from both the low- and high-risk groups was comparable, the high-risk group data was calculated from the final pathological result. If using frozen sections, the data of CA125 may be better than the Mayo Clinic prediction system. Importantly, serum CA125 could be obtained preoperatively and used when counseling patients about the potential risks and benefits of lymphadenectomy, or referring high-risk patients to specialized gynecologic oncologists for comprehensive surgical staging, including systematic lymphadenectomy. From the survival analysis, the disease-free survival of patients with CA125 ≤ 25 U/mL was longer than those with CA125 > 25 U/mL. Therefore, 25 U/mL of CA125 may be a helpful marker for oncologists to decide whether a lymphadenectomy should be performed on patients with clinical stage I endometrial cancer.

The normal CA125 level in postmenopausal women is <15 U/mL, which is significantly lower than that found in premenopausal women [17,18]. Chao [14] proposed the use of an age-adjusted cutoff for preoperative CA125 levels to improve the prediction of lymph node metastases in patients with endometrial cancer. In current study, the median value of CA125 was also related to age and menopause. Therefore, we examined whether age and menopause influence the value of preoperative CA125 in predicting lymph node metastasis. The best cutoff was different for premenopausal or ≤ 50 years and menopausal or > 50 years patients. The best cutoff of CA125 for predicting the lymph node metastasis rose to 30 U/mL from 25 U/mL in patients ≤ 50 years of age or with premenopause. Consequently, if preoperative levels of serum CA125 are used in the clinic, both age and menopause should be considered.

CA125 was first reported as a circulating antigen in women with epithelial ovarian cancer. Therefore, it should be a good predictor for adnexal involvement in endometrial cancer. In agreement with previous studies [15,19,20], our results also demonstrated that higher serum CA125 levels were associated with adnexal involvement in endometrial cancer. The best cutoff value of serum CA125 level of 30 U/mL was with 84.6% of sensitivity, 84.3% of specificity in predicting adnexal involvement in endometrial cancer. In patients with clinical stage I, 30 U/mL of preoperative serum CA125 was also with 81% of sensitivity, 78.4% of specificity in predicting adnexal involvement.When we focused on premenopausal patients with clinical stage I, the best cutoff value of serum CA125 level of 30 U/mL was with 80% of sensitivity, 73.2% of specificity in predicting adnexal involvement. Thus, from the current study, 30 U/mL of serum CA125 may be helpful in preoperative counseling for young patients with endometrial cancer who want to preserve their ovaries.

Complete cytoreduction has been shown to improve median survival in advanced stage endometrial cancer. However, the difficulty of identifying micrometastases, which are invisible to the naked eye, at the time of surgery limits the effectiveness of this operation. In our study, higher serum levels of CA125 were associated with extrauterine metastasis including lymph node metastasis, distant metastasis and positive peritoneal cytology in endometrial cancer. In patients with clinical stage I, 30 U/mL of preoperative serum CA125 was with 74.3% of sensitivity and 81.9% of specifity in predicting extrauterine metastasis. Thus, from the current study, 30 U/mL of serum CA125 may be helpful to determine which patients will benefit from a complete cytoreduction.

The following advantages of the current study should be acknowledged. First, our study was the largest retrospective study on the value of preoperative serum CA125 in the optimal surgical management of endometrial cancer. Second, in inclusion criteria, we excluded the patients who might have had other medical co-morbidities that contributed to elevated serum CA125 levels, independent of extrauterine disease. Third, we focused on the patients with cinlical stage I, where the decision for systemic lymphadenectomy and adnexectomy in premenopausal patients is not definitive. Fourth, in calculating the best cutoff of CA125 for lymph node metastasis, we only calculated the patient with only lymph node metastasis, which can omit the influence from the adnexal involvement, distant metastasis and positive peritoneal cytology. Together, these help guarantee more precise results.

However, the current study has some limitations. First, this was a retrospective study, and the intraoperative and postoperative management of patients with an elevated level of serum CA125 was not different from those of healthy individuals. Second, there was a selection bias, as 18.8% of 1,226 patients were excluded owing to a lack of preoperative serum CA125 levels, incomplete staging operation, or loss of follow up. Third, it was a single-center study. Therefore, to confirm these results, a future large-size, multi-center study is needed.

## Conclusions

The main purpose of this analysis was to evaluate if preoperative serum CA125 was helpful for gynecologic oncologists to determine the surgical management in endometrial cancer, particularly whether preoperative CA125 serum levels could indicate if a lymphadenectomy was required for clinical stage I patients.We found that preoperative serum CA125 was a good predictor of lymph node metastasis for patients with endometrial cancer, especially patients with clinical stage I. In premenopausal patients with clinical stage I, preoperative serum CA125 was also helpful for those patients who seek to preserve their ovaries. If preoperative serum CA125 was too high in patients with clinical stage I, complete cytoreduction could be considered. Therefore, preoperative serum CA125 is an important predictor for patients with endometrial cancer and it should be taken into consideration when surgical management is determined, especially if a lymphadenectomy should be undertaken in patients with clinical stage I.

## Abbreviations

FIGO: International Federation of Gynecology and Obstetrics
NCCN: National Comprehensive Cancer Network
ROC: Receiver operating characteristic
CA125: Cancer antigen 125
BMI: Body max index
